# Time Varying Apparent Volume of Distribution and Drug Half-Lives Following Intravenous Bolus Injections

**DOI:** 10.1371/journal.pone.0158798

**Published:** 2016-07-12

**Authors:** Carl A. Wesolowski, Michal J. Wesolowski, Paul S. Babyn, Surajith N. Wanasundara

**Affiliations:** 1 Department of Medical Imaging, College of Medicine, University of Saskatchewan, Saskatoon, Saskatchewan, Canada; 2 Department of Radiology, Memorial University of Newfoundland, St. John's, Newfoundland, Canada; Cleveland Clinic, UNITED STATES

## Abstract

We present a model that generalizes the apparent volume of distribution and half-life as functions of time following intravenous bolus injection. This generalized model defines a time varying apparent volume of drug distribution. The half-lives of drug remaining in the body vary in time and become longer as time elapses, eventually converging to the terminal half-life. Two example fit models were substituted into the general model: biexponential models from the least relative concentration error, and gamma variate models using adaptive regularization for least relative error of clearance. Using adult population parameters from 41 studies of the renal glomerular filtration marker ^169^Yb-DTPA, simulations of extracellular fluid volumes of 5, 10, 15 and 20 litres and plasma clearances of 40 and 100 ml/min were obtained. Of these models, the adaptively obtained gamma variate models had longer times to 95% of terminal volume and longer half-lives.

## Introduction

The apparent volume of distribution (*V*_*d*_) is an important pharmacokinetic parameter that relates drug plasma concentrations to the amount of drug in the body and is important for drug loading dose and maintenance dose calculations [1,2,3]. Following an intravenous bolus of drug, the volume of distribution of a drug varies with time. This redistribution occurs in two distinct phases with very different time scales, a vascular phase and a washout or dilution phase. During the vascular phase, a drug mixes throughout the blood’s plasma volume with a time scale of seconds or minutes. During the dilution phase, hydrophilic drugs distribute into the body’s interstitial fluids with a time scale of hours or days. While most drugs are actively redistributing from plasma into the body tissues, the decrease of plasma concentrations is largely due to that redistribution as opposed to actual drug elimination [2]. In the following, we assume first order kinetics, i.e., that drug elimination is proportional to its concentration, which is the most common drug kinetic. The most commonly calculated volumes of distributions are the apparent volume of drug distribution immediately after bolus intravenous injection, i.e., at time zero, (*V*_0_), the terminal apparent volume of distribution following bolus intravenous administration (*V*_area_) and the expected volume of distribution, called *V*_E_ here, and often called *Vss* in the literature. *V*_*SS*_ for a constant infusion experiment is the terminal apparent volume of distribution, analogous to *V*_area_ in a bolus model [2,3]. For bolus experiments, in place of *V*_*SS*_ we use the term *V*_E_ for the expectation of a physical volume of distribution of drug, and unlike *V*_*SS*_, *V*_E_ is invariant between constant infusion and bolus experiments.

Time varying apparent volume of distribution models based on exponential washout models for plasma concentration, have been developed previously [2,4,5]. Niazi [4] developed a temporally variable, apparent volume of distribution model for sum of exponential (SET) functions based on conservation of mass. This variable volume model implies an explicit relationship between redistribution and the rate of volume of drug distribution expansion in time. Such a model specifies when the drug volume reaches a particular size relative to its terminal apparent volume of distribution. In so doing, such a variable volume model could potentially provide unanticipated new information about time based tissue drug effects for tissue metabolism and/or eliminations. This type of model also has implications for effects of drugs on body tissues, i.e., therapeutic, toxic, or radiation exposure effects [3].

A variable volume model could be used to calculate the optimal instantaneous dose that will produce a desired concentration at the effector site without an overload of the drug. Here we present a general time-dependent apparent volume of distribution model, based on mass conservation, which could be used with almost any concentration washout fit function. To investigate how the implications of the general model relate to specific fit function models, we substitute biexponentials (E2) and gamma variates (GV) [6,7,8] into the general washout model.

## Theory

### The variable volume of distribution

Plasma clearance (*CL*) is a drug’s elimination rate, *M*′(*t*), divided by its corresponding plasma concentration, *C*(*t*), at any time *t* [9],

$$CL=-\frac{M\prime \left(t\right)}{C\left(t\right)},$$ (1)

where *M*(*t*) is total drug mass in the body at time *t* and the prime indicates differentiation. Rearrangement of Eq (1) gives the drug’s elimination rate,

M′(t)=−CL C(t). (2)

Integrating both side of Eq (2) from time 0 to *t* gives mass remaining in the body at time *t* to be

$$M\left(t\right)=M\left(0\right)-CL\int_{0}^{t}C\left(\tau \right)d\tau =CL\int_{t}^{\infty }C\left(\tau \right)d\tau ,$$ (3)

where *M*(*t→∞*) = 0.When *t* = 0 and *M*(*t = 0*) = *D*, the dose, from equality of the left and right hand functions of Eq (3), one solves for *CL* to obtain the well-known area under the curve, *AUC*, definition of *CL*,

$$CL=\frac{D}{\int_{0}^{\infty }C\left(\tau \right)d\tau }.$$ (4)

Conservation of mass for a concentration in an apparent (homogenous plasma concentration) volume of distribution, *V*_*d*_(*t*), at time *t*, is given by

$$M(t)=V_{d}(t)\;C(t).$$ (5)

The typical pharmacokinetic model specifies the washout of an impulse-response. The impulse is the entire initial dose *M*(0) = *D* distributed in some initial volume no matter how small. *M*(*t*) is monotonic non-increasing (mass conservation) and *V*_*d*_(*t*) monotonic increasing. Thus, *C*(*t*) is monotonic decreasing, and has a maximum value, no matter how large, at time is zero. Substitution of Eq (5) into Eq (3) yields the volume of distribution at any time *t* as

$$V_{d}\left(t\right)=\frac{CL}{C(t)}\int_{t}^{\infty }C\left(\tau \right)d\tau .$$ (6)

### Expected volume of distribution

Expected volume of distribution (*V*_E_) is the expected value of physiological volume of distribution of drug in the plasma and tissues of a concentration model. *V*_E_ inherits the statistical properties for expectation from its distribution density function model, *p*(*t*),

$$V_{\text{E}}=CL\text{ E}\left[T\right]$$ (7)

For example, if the distribution density function, *p*(*t*), has a mean value expectation, $\text{E}\left[T\right]=\bar{t}$, we can then calculate the expected volume of drug distribution from that mean, which for a time series is called a mean residence time ($MRT=\bar{t}$) [10,11]. *MRT* is the average value or first moment of a time-series density function.

$$MRT=\bar{t}=\overset{\infty }{\underset{0}{\int }}\;t\;dP=\overset{\infty }{\underset{0}{\int }}\;t\;p(t)\;dt=\overset{\infty }{\underset{0}{\int }}\;t\frac{C(t)}{\int_{t=0}^{\infty }C\left(t\right)dt}dt=\frac{\int_{0}^{\infty }t\;C\left(t\right)dt}{\int_{0}^{\infty }C\left(t\right)dt},$$ (8)

where *P* is the cumulative density function of *p*. To be useful, Eq (8) assumes that drug elimination rate is first order, i.e., that elimination is not dose dependent [12]. If a density function has no moments, *MRT* is undefined but there may still be a location that characterizes the data, for example, a Cauchy distribution has a stable median[*T*]. Indeed, for a distribution the median can be a better measure of location than the expected value, e.g., for some values of the beta distribution. For some density functions, E[*T*], is indeterminate for heavier tails, e.g., for the Pareto distribution.

We suggest that E[*T*] for a constant infusion experiment is also from *p*(*t*), and *V*_E_ is from Eq (7). However, *p*(*t*) is the impulse-response to an instantaneous bolus, i.e., to a Dirac delta, and to obtain a *p*(*t*), we account for the integration of constant infusion as $P\left(t\right)=\int_{0}^{t}p\left(\tau \right)d\tau$, the cumulative density of *p*(*t*). We then suggest fitting $\frac{D_{\text{R}}}{CL}P\left(t\right)=C_{\text{SS}}\;P\left(t\right)$ to constant infusion data, where *D*_R_ is the dose rate of constant infusion, *D*_R_*/CL* is the terminal concentration of the infusion experiment usually called *C*_SS_. Once we have a *p*(*t*), its derivative is *p*(*t*).

### Half-life of the drug, *t*_1/2_

The half-life of any drug’s mass in the body at time *t* can be written as

$$t_{1/2}\text{-}M(t)=\frac{\text{ln}2}{-\frac{d}{dt}\text{ln}M(t)}=-\text{ln}2\;\frac{M(t)}{M\prime (t)},$$ (9)

where for a marker cleared only by the kidney this half-life corresponds to the urinary clearance half-life. Substituting Eqs (2 & 5) into Eq (9) yields an expression for half-life of a variable apparent volume

$$t_{1/2}\text{-}M(t)=\frac{\text{ln}2}{CL}V_{d}(t).$$ (10)

However, the urinary clearance of kidney marker half-life is not, as a function of time, the same as the half-life of plasma concentration. To find the latter, we first substitute Eq (1) into the derivative of Eq (5) and rearrange terms to obtain

$$\frac{C\prime \left(t\right)}{C\left(t\right)}=-\frac{CL+V_{d}\prime \left(t\right)}{V_{d}\left(t\right)},$$ (11)

which allow the half-life of plasma concentration as a function of time to be written as

$$t_{1/2}\text{-}C{\left(t\right)}_{}=\frac{\text{ln}2}{-\frac{d}{dt}\text{ln}C(t)}=-\text{ln}2\;\frac{C(t)}{C\prime (t)}=\frac{\text{ln}2\;}{CL+V_{d}\prime \left(t\right)}V_{d}\left(t\right).$$ (12)

Given that *V*_*d*_(*t→∞*) = *V*_area_ (apparent volume at terminal phase), and *V*_*d*_′(*t→∞*) = 0, a unique terminal half-life, *t*_1/2_(∞), is the limiting value for both Eqs (10) and (12), i.e., for mass (e.g., renal) clearance and plasma concentration half-lives

$$t_{1/2}\left(\infty \right)=t_{1/2}\text{-}M\left(\infty \right)=t_{1/2}\text{-}C\left(\infty \right)=\frac{\text{ln}2}{CL}V_{\text{area}},$$ (13)

where at all other times, comparing Eqs (10 & 12), *t*_1/2_-*M*(*t*) > *t*_1/2_-*C*(*t*), such that before the terminal half-life becomes established, plasma concentration dilutes faster than mass clearance. To show this, Eqs (2, 5 & 11) are combined and terms rearranged to yield an explicit relationship between plasma dilution rate and mass clearance rate,

$$\;-\frac{C\prime (t)}{C(t)}=\;-\frac{M\prime (t)}{M(t)}+\frac{\;V_{d}\prime \left(t\right)}{V_{d}\left(t\right)},$$ (14)

such that the relative rate of plasma dilution is the relative rate of mass clearance plus redistribution, where redistribution is the relative rate of volume dilution. To see the effect sizes and their timing, we next substitute specific fit functions into the general model equations above, and later apply them to subject data.

### Exponential solutions to the variable volume and half-life equations

In the exponential model, plasma concentration is given by sum of exponentials,

$$C_{\text{exp}}\left(t\right)=\overset{n}{\underset{i=1}{\sum }}c_{i}e^{-\lambda_{i}t},\;C_{\text{exp}}\prime \left(t\right)=-\overset{n}{\underset{i=1}{\sum }}\lambda_{i}c_{i}e^{-\lambda_{i}t}:\;c_{i}>0\text{ and }\lambda_{i}>0,$$ (15)

where *c*_*i*_ and *λ*_*i*_ are the coefficients of *i*^th^ exponential term. Substituting the sum definition of *C*_exp_(*t*) from Eq (15) into Eq (3) yields a sum of exponential terms solution to drug mass remaining in the body at time *t*,

$$M\left(t\right)=CL\overset{n}{\underset{i=1}{\sum }}\frac{c_{i}e^{-\lambda_{i}t}}{\lambda_{i}}.$$ (16)

Similarly, substitution of the *C*_exp_(*t*) sum, Eq (15) into the general form for *V*_*d*_(*t*),Eq (6), yields,

$$V_{d}\left(t\right)=\frac{CL}{\sum_{i=1}^{n}c_{i}e^{-\lambda_{i}t}}\overset{n}{\underset{i=1}{\sum }}\frac{c_{i}e^{-\lambda_{i}t}}{\lambda_{i}}.$$ (17)

Substitution of *t* = 0,∞ into this equation allows us to specify the initial (*V*_0_) and final apparent volumes of distribution *V*_area_,

$$V_{0}=V_{d}\left(t=0\right)=\frac{CL}{\sum_{i=1}^{n}c_{i}}\overset{n}{\underset{i=1}{\sum }}\frac{c_{i}}{\lambda_{i}}=\frac{D}{\sum_{i=1}^{n}c_{i}},$$ (18)

$$V_{\text{area}}=V_{d}\left(t\to \infty \right)=\frac{CL}{\lambda_{n}}.$$ (19)

In a compartmental model from sums of exponential terms, *V*_0_ is identical to the volume of the central compartment *V*_c_.

Substitution of Eq (15) into Eq (4) yields the *CL* for SET model,

$$CL=\frac{D}{\sum_{i=1}^{n}\frac{c_{i}}{\lambda_{i}}}.$$ (20)

Substitution of Eq (15) into Eqs (7 & 8) and performing the integration yields a steady-state volume of distribution (*V*_*SS*_) for SET models, which in our more general context, we call *V*_E_,

$$V_{\text{E}}=CL\frac{\sum_{i=1}^{n}\frac{c_{i}}{\lambda_{i}^{2}}}{\sum_{i=1}^{n}\frac{c_{i}}{\lambda_{i}}}.$$ (21)

Substituting of the SET *V*_*d*_(*t*), Eq (17), into general half-life Eq (10) yields the mass clearance half-life for SET functions at time *t*,

$$t_{1/2}\text{-}M\left(t\right)=\frac{\text{ln}2}{\sum_{i=1}^{n}c_{i}e^{-\lambda_{i}t}}\overset{n}{\underset{i=1}{\sum }}\frac{c_{i}e^{-\lambda_{i}t}}{\lambda_{i}}.$$ (22)

The mass clearance half-life would equal the renal clearance half-life for a *GFR* marker.

### A gamma variate solution to the variable volume and half-life equations

Regularized GV functions are of interest because they have been previously shown to require one-half the sampling time (4 h) needed for numerical integration (8 h) to obtain precise and accurate CL-values in a large retrospective series [6]. The plasma concentration as a function of time can be modelled by gamma variate (GV) function,

$$C_{\text{GV}}\left(t\right)=Kt^{\alpha -1}e^{-\beta t},\;C_{\text{GV}}\prime \left(t\right)\text{=}\left(\frac{\alpha -1}{t}-\beta \right)C_{\text{GV}}\left(t\right):\;0<\alpha <1\text{ and }\beta >0,$$ (23)

where *α*, *β* and *K* are the three parameters of a GV function. Note that *α* < 1 is not a constraint, and there is so far only one published method of consistently obtaining *α* < 1 values without using constraints [6,7,8,13]. Substitution of *C*_GV_(*t*) Eq (23) into general Eq (3) yields the gamma variate solution to drug mass remaining in the body at time *t*,

$$M\left(t\right)=CL\frac{K}{\beta^{\alpha }}\Gamma \left(\alpha ,\beta t\right)=D\frac{\Gamma \left(\alpha ,\beta t\right)}{\Gamma \left(\alpha \right)},$$ (24)

where Γ(*α*) and Γ(*α*, *βt*) are respectively, the gamma function and the upper incomplete gamma function. Substitution of *C*_GV_(*t*) Eq (23) into Eq (6) and performing the indicated integration yields

$$V_{d}\left(t\right)=\frac{CL}{\beta }\frac{\text{Γ}(\alpha ,\beta t)}{{\left(\beta t\right)}^{\alpha -1}e^{-\beta t}};\;0<\alpha <1.$$ (25)

This equation is monotonically increasing only when *α* < 1, which is not a constraint for obtaining *α*-values, rather the meaning is that the upper limit for physicality of *α*-values is one. In that case, the initial (*V*_0_) and final (*V*_area_) apparent volume of distribution in the limit as time goes to 0 and to infinity are respectively given by

$$V_{0}=V_{d}\left(t\to 0\right)=0,$$ (26)

$$V_{\text{area}}=V_{d}\left(t\to \infty \right)=\frac{CL}{\beta }.$$ (27)

Substitution of Eq (23), the *C*_GV_(*t*), into general Eq (4) and performing the indicated integration yields an expression for *CL* of a GV model

$$CL=\frac{D\;\beta^{\alpha }}{K\text{ Γ}(\alpha )}.$$ (28)

Substitution of Eq (23) into Eqs (8 & 7) yields the physical volume of distribution for GV model,

$$V_{\text{E}}=CL\frac{\alpha }{\beta }.$$ (29)

Note that from this and Eq (27), the GV model *V*_area_ = *αV*_E_. Moreover, from Eqs (24, 25 & 27)

$$\underset{t\to \infty }{\text{lim}}\frac{M\left(t\right)}{V_{\text{area}}-V_{d}\left(t\right)}\;=\;\frac{K}{\beta^{\alpha -1}}\underset{\beta t\to \infty }{\text{lim}}\frac{\Gamma \left(\alpha ,\beta \;t\right)}{1-\frac{\Gamma \left(\alpha ,\beta \;t\right)}{{\left(\beta \;t\right)}^{\alpha -1}e^{-\beta \;t}}}\;=0,$$ (30)

This limit exists except for *α* = 1, while *β* > 0. This latter does not occur for the GV solutions used here, which yield *β*→0+ when α→1−, i.e., concentration flatlines rather than become exponential [6]. When 0 < *α* < 1, *V*_d_ is monotonic increasing and denominators of Eq (30) are positive valued. [Disp-formula pone.0158798.e034] insures that *M*(*t*) < *ε* for *V*_*d*_(*t*_*ε*_ < *t* < *T*), where *T* is a sufficiently large but finite time. Thus, *V*_*d*_(*t*) becomes as mass depleted as desired for *t* sufficiently large but less than some *T*-large, i.e., before *V*_*d*_ converges to *V*_area_ at infinite time. Consequently, the single bolus experiment GV model’s drug depleted physical volume is written *V*_E_. That is, the GV washout model steady state is trivial and empty. The restriction on Eq (30) is that the GV not be an exponential. Readers who use hazard rates to *classify* tail heaviness of distributions may find this confusing. Hazard rate classification of tail heaviness is inexact and actual terminal tail areas compare as survival functions. From survival function ratios, gamma distributions with *α* > 1 have lighter than exponential tails, and for *α* < 1, i.e., the general case here, gamma distributions tails are heavier than exponential.

Substituting *V*_*d*_(*t*) from Eq (23) into Eq (10) yields the half-life of the drug mass remaining in the body

$$t_{1/2}\text{-}M(t)=\frac{\text{ln}2}{\beta }\frac{\text{Γ}(\alpha ,\beta t)}{{\left(\beta t\right)}^{\alpha -1}e^{-\beta t}}.$$ (31)

## Methods

To test the time varying volume of distribution model, concentrations verses time curves for four *V*_E_ values (10, 15, 20 and 25 L) at *CL* of 40 and 100 ml/min were simulated for biexponential (E2) and the gamma variate (GV) models. E2 and GV parameters were computed by using prior published data as follows.

Data were used here from a prior study of 41 plasma concentration time samplings following intravenous bolus ^169^Yb-DTPA (ytterbium diethylenetriaminepentaacetate) [14]. In this population, patients were given an antecubital IV bolus injection of 1.85 MBq of ^169^Yb-DTPA. Eight blood samples were taken at 10, 20, 30, 45, 60, 120, 180 and 240 min after injection. Plasma clearance *CL* and *V*_E_ for E2 and GV functions were calculated for all 41 patients. E2 functions were random search fit using relative-concentration weighted regression that preferentially fits longer times giving better results than from the use of ordinary least squares [15,16]. The Tk-GV algorithm uses Tikhonov regularization to increase covariance between the GV function and the time-samples by minimizing the *AUC* error over the entire interval from *t* = 0 to ∞. This minimizes the relative error of plasma clearance [6]. The GV functions’ three parameters were obtained from the Tk-GV method. An important point is that the Tk-GV method uses adaptive smoothing and without this feature the resulting PK parameter GV model results will be erratic [6,8]. A Windows compatible Tk-GV software application is available to individual researchers (i.e., not institutions) free of charge from the corresponding author.

Parameters for desired *V*_E_ and *CL* values were obtained by interpolating parameters obtained from above fitted curves. Using computed parameters, volume of distribution, drug mass remaining in the body and drug half-lives as a function of time were plotted for four *V*_E_-values at *CL* of 100 and 40 ml/min. We have ignored some of the different morphological implications of the different modelling in order to generate comparison curves. That is, *CL* and *V*_E_ were assigned the same specific values, e.g., 100 ml/min and 20 litres, respectively, for both the E2 and Tk-GV models. For actual cases, that will not occur within the same hypothetical subject, and the same case between model *CL-* and *V*_E_-values differ slightly. A statistical analysis of the parameters for the reference population used appears in the Result Section below.

## Results

### Biexponential (E2) model

**Fig 1:**
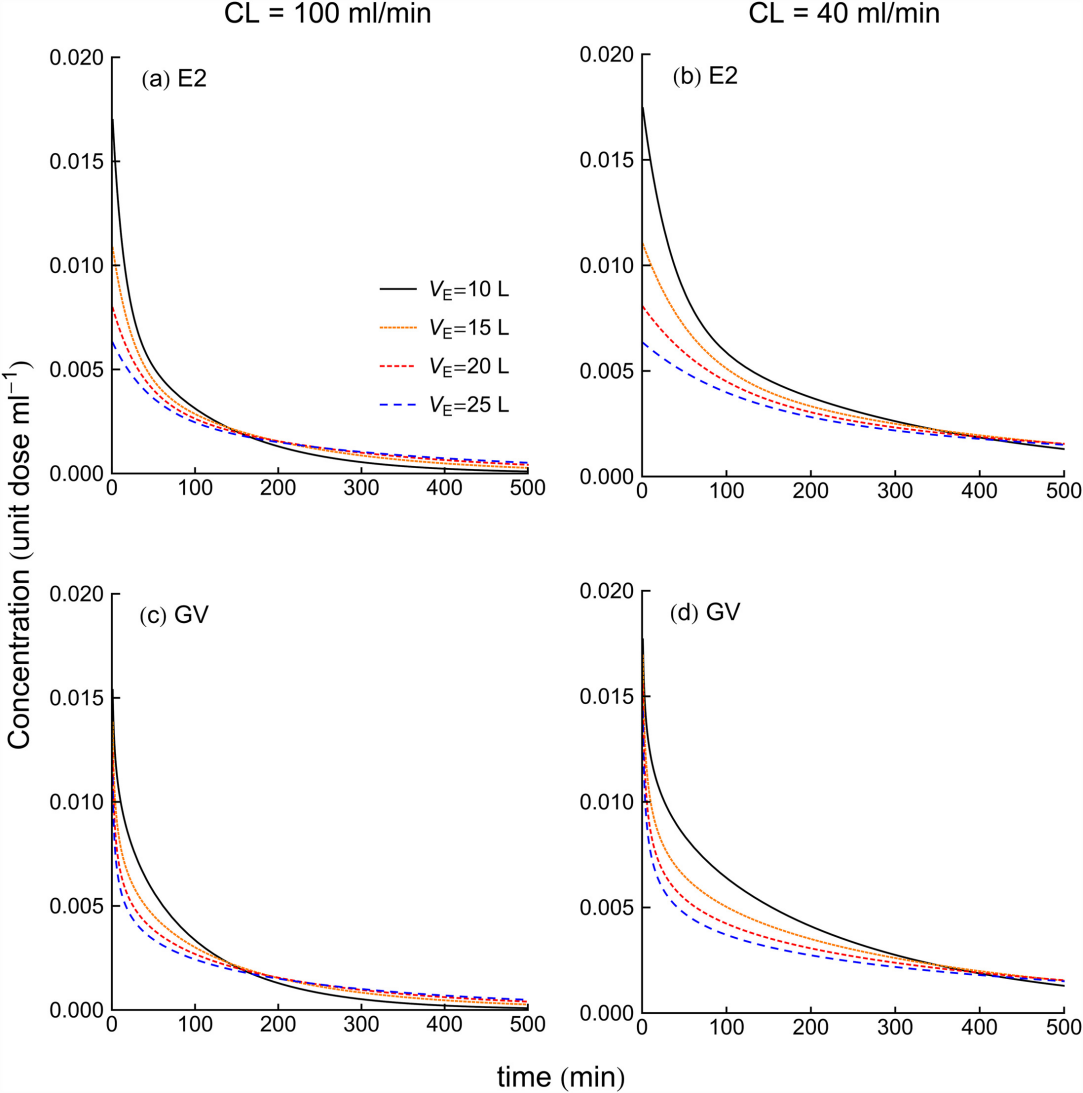
Concentration versus time curve for E2 and GV models for four *V*_E_ values at *CL* of 100 ml/min (left panel) and 40 ml/min (right panel).

**Fig 2:**
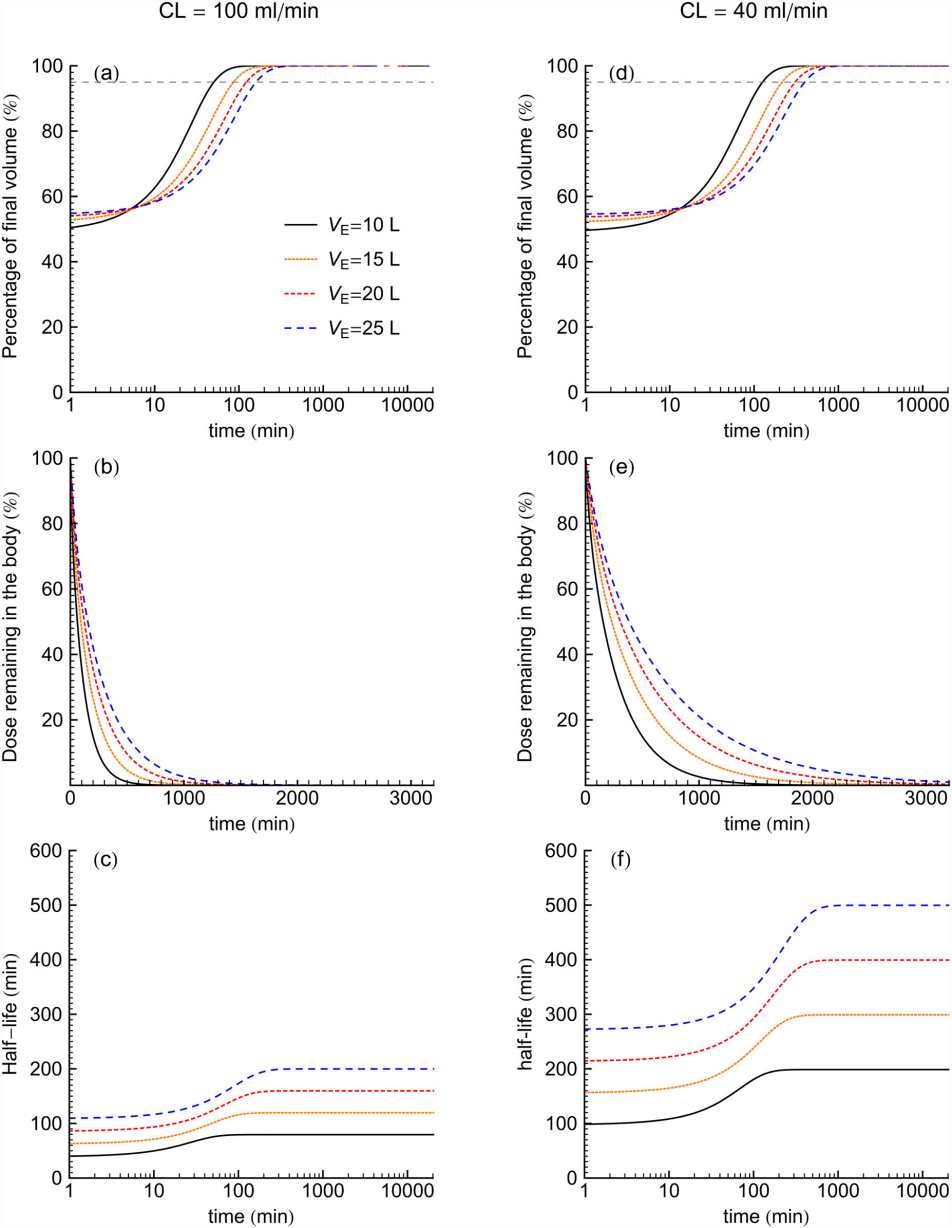
For E2 models for four *V*_E_ values at *CL* of 100 ml/min (left panel) and 40 ml/min (right panel), the percentage of terminal apparent volume, percent dose mass in time and half-life of the drug as a function of time. Dashed horizontal line indicates 95% of final volume.

Fig 1a and 1b illustrate concentration verses time curves for E2 models for four *V*_E_ values at *CL* of 100 and 40 ml/min, respectively. Fig 2 shows the volume of distribution, percentage dose remaining in the body and half-lives for the E2 model as functions of time for the four *V*_E_ values at *CL* of 100 and 40 ml/min. The time to achieve *V*_area_ (Fig 2a and 2d) is increased with the increase of volume of distribution. On the other hand, Fig 2b and 2e show that dose in the body reduces at a faster rate for higher *CL*. In both Fig 1c and 1f, half-life is increased with the increase of the fluid volume. Furthermore, comparing same two figures (Fig 1c and 1f) at each *V*_E_ shows that decrease of clearance tends to increase of the half-life.

### Gamma variate (GV) model

**Fig 3:**
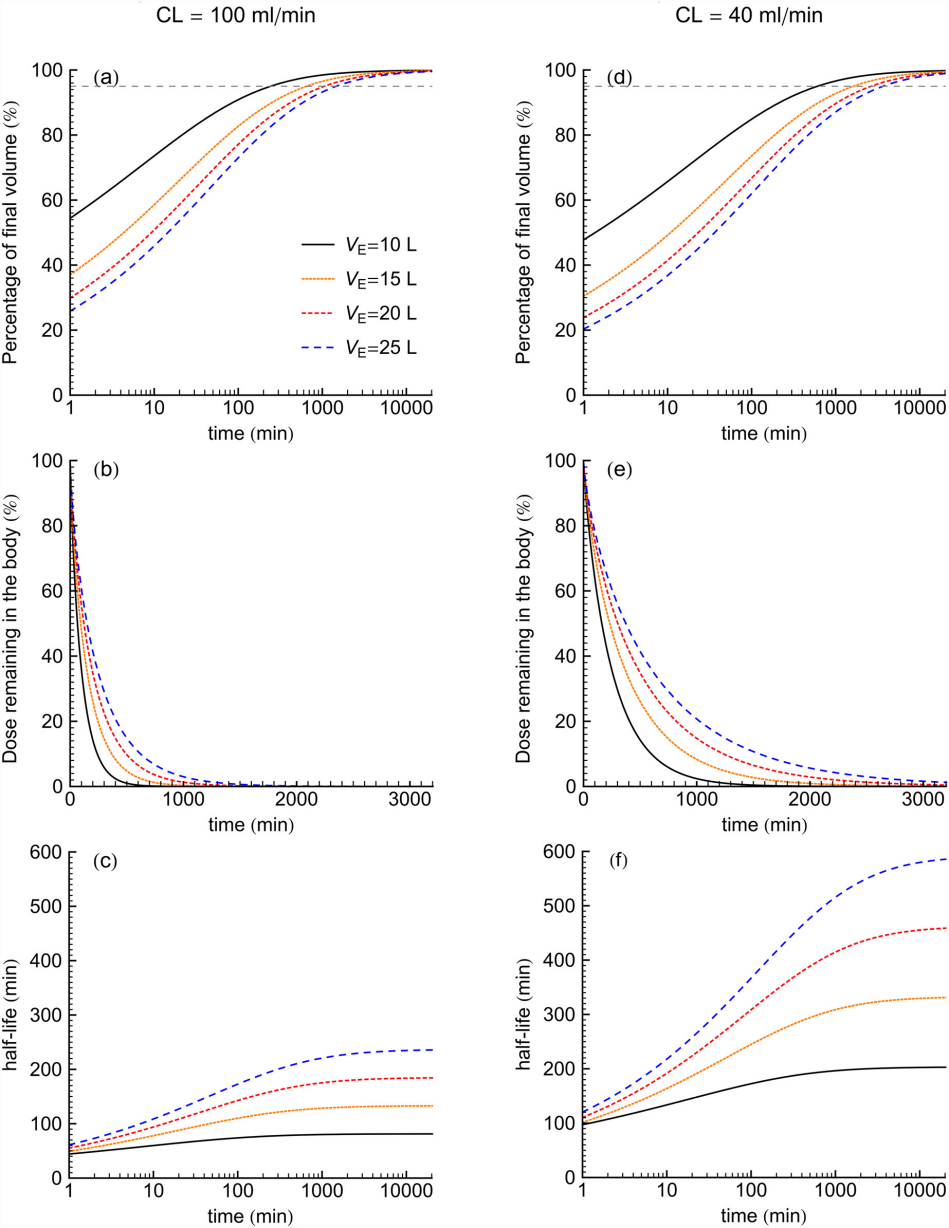
For GV models for 4 *V*_E_ values at *CL* of 100 ml/min (left panel) and 40 ml/min (right panel), the percentage of terminal apparent volume, percent dose mass in time and half-life of the drug as a function of time. Dashed horizontal line indicates 95% of final volume.

Fig 1c and 1d illustrate concentration verses time curves for GV models for four *V*_E_ values at *CL* of 100 and 40 ml/min, respectively. Fig 3 shows the volumes of distribution, percentage dose remaining in the body and half-life calculated from GV model as a function of time. When we consider the time to achieve 95% of *V*_area_, increase of *V*_E_ increases the time to achieve 95% of *V*_area_. Fig 3b and 3e show that decreased dose remaining in the body occurred sooner for the larger *CL*-values, while smaller *CL*-value patients took more time to clear drugs from the body with an additional delay to achieve a given mass excretion seen with the increase of *V*_E_. Fig 3c and 3f show that half-lives were longest for the smaller *CL*-values. Furthermore, half-life increased for increasing *V*_E_.

For comparison of the E2 and GV methods, Table 1 lists the time for *V*_d_ to reach 95% of V_area_ and drugs terminal half-life following intravenous boluses. Both time values shown in the Table 1 increase with the increase of the *V*_E_ as well as with the decrease of the *CL*. Even though we selected equal *CL* and *V*_E_ parameter values for our demonstration E2 and GV models, the GV models still predicted longer times for *V*_d_ to reach 95% of V_area_ as well as longer terminal half-lives.

**Table 1:** Time to achieve apparent volume of distribution to 95% of *V*_area_ after the intravenous bolus of the drug and terminal half-life of the drug from E2 and GV models.

*V*_E_ (L)	10		15		20		25	
	E2	GV	E2	GV	E2	GV	E2	GV
CL = 100 ml/min								
time for *V*_d_ to reach 95% *V*_area_ (min)	50	242	86	646	123	1053	160	1460
terminal half-life of drug	80	81	120	133	160	185	200	238
CL = 40 ml/min								
time for *V*_d_ to reach 95% *V*_area_ (min)	126	604	215	1616	307	2632	399	3650
terminal half-life of drug	200	203	300	333	400	462	500	592

For same-case concentration data, it has been shown that the E2 and GV (Tk-GV) models will predict significantly, if slightly different *CL* and *V*_E_ values [6,7,8]. To see the errors for these differences for the subject cases herein, bootstrap was used to avoid exaggerating the E2 model 95% confidence interval (CI) and standard deviation (SD) results that would occur if one spuriously assumed normal distribution conditions especially for the early exponential function’s population parameters, which latter were found to be not normally distributed. For the Tk-GV method, the resulting gamma variate parameters were quasi-normally distributed, which allowed for the routine Tk-GV program SD-values to be used for calculating the CIs from their more normal distribution properties. Table 2 shows that the CIs are larger for E2 than for Tk-GV.

**Table 2:** Pharmacokinetic parameters from the weighted biexponential (E2) and the Tk-GV models. Comparable measures boxed.

E2	*c*_1_	*λ*_1_	*c*_2_	*λ*_2_	*CL*	*V*_E_	*t*_1/2_
2.5% tail of median ^a^	0.00413	0.03321	0.00470	0.00334	61.0	13.21	116
Median ^b^	0.00484	0.05020	0.00555	0.00459	79.0	15.48	151
97.5% tail of median ^a^	0.00891	0.10517	0.00611	0.00596	82.8	17.39	208
Median CV ^a^	17.3%	27.6%	5.5%	11.1%	5.5%	6.3%	- ^c^
Minimum ^b^	0.00207	0.01362	0.00090	0.00023	2.6	7.18	67
Maximum ^b^	0.05185	0.21546	0.01260	0.01040	166.4	26.46	2999
Tk-GV		ln*K*	*α*	*β*	*CL*	*V*_E_	*t*_1/2_
2.5% tail of median ^b^		-4.456	0.7264	0.00301	70.1	15.07	166
Median ^b^		-4.288	0.7556	0.00360	76.1	16.15	193
97.5% tail of median ^b^		-4.143	0.8027	0.00418	79.7	17.26	230
Median CV ^b^		-1.8%	2.7%	8.3%	4.1%	2.7%	- ^c^
Minimum ^b^		-5.364	0.5945	0.00011	1.2	7.40	76
Maximum ^b^		-3.386	0.9895	0.00908	157.6	31.12	6559
Median SD ^b^		0.071	0.0222	0.00027	2.3	0.47	-

SD is standard deviation and CV is coefficient of variation or SD divided by the mean.

^a^ Median of 41 cases with each case result from the distribution of 1000 bootstrap simulations attempts per case.

^b^ Result from 41 cases and not requiring simulation.

^c^ Reciprocal normal. Comparative CV values can be taken from *λ*_2_ and *β* CVs.

Some of the (many) parameters that were significantly different between models are summarized in Table 3. Note that *V*_E_, *V*_area_, *t*_1/2_ and *MRT* are greater for Tk-GV than for E2, and that *CL* and CV(*V*_E_) are lesser and as well *β* < *λ*_2_. Thus, although for easy comparison of families of curves in the figures, *CL* and *V*_E_, were assigned the same specific values for both the E2 and Tk-GV models, we were, in effect, contrasting two hypothetical subjects with slightly different body morphologies.

**Table 3:** Wilcoxon tests with median parameter comparisons.

Parameter	Tk-GV	Result (*H*_1_)	E2	*p*-one tail	Significant
*CL*	76.1	<	79.0	<0.0001	Yes
*β*,*λ*_2_	0.00360	<	0.00459	<0.0001	Yes
CV(*V*_E_)	2.7%	<	6.3%	<0.0001	Yes
*V*_E_	16.15	>	15.48	0.0003	Yes
*V*_area_	21.46	>	16.28	<0.0001	Yes
*t*_1/2_	193	>	151	<0.0001	Yes
*MRT*	213	>	194	<0.0001	Yes

Fit quality for the Tk-GV and E2 models has been presented elsewhere for this adult data set—see Fig 1 and surrounding text of [6]. Herein, normalized covariances, i.e., correlations, between the logarithms of the time-samples and of each model evaluated at those times were considered indexed to the fidelity of trending between the data and the two models. These correlations had a median *r*-value for Tk-GV of 0.99840 and of 0.99832 for E2, with a two-tailed Wilcoxon *p* = 0.27 suggesting no significant difference in the overall quality of how each model trended with the data. However, even comparing *r*-values is somewhat misleading. The Tk-GV algorithm smooths its model curve to stabilize *AUC* on the 0 to infinite time interval, which time interval is unrelated to calculation of its *r*-value with the data, whereas the weighted-E2 models were fitted to concentration in a fashion numerically identical to the time-sample treatment used to compute *r*-values, such that the lack of significant improvement of *r*-values for weighted-E2 versus Tk-GV fitting does not increase our confidence in E2 model behaviour; weighted-E2 modelling was given an unfair advantage to outperform Tk-GV modelling, and did not do so.

## Discussion

In both the biexponential (E2) and gamma variate (GV) variable volume models, smaller values of *V*_E_ approached 95% of their terminal *V*_*d*_-values sooner than those with relatively greater *V*_E_ values, see Table 1. Figs 2b and 3b show that an increased fluid volume was associated with prolonged drug-levels at late time. These results are generally consistent with the known delays in redistribution from excess body fluid and in advanced renal insufficiency [7,17,18]. Furthermore, both the E2 and GV models demonstrated that drug body burden decreased sooner for studies having larger *CL*-values. Figs 2c, 2f, 3c and 3f show that for both model types, longer half-lives occurred for larger *V*_E_ value patients in both *CL* of 100 and 40 ml/min. Drug elimination depends on the amount of drug delivered to the organs where excretion occurs. Therefore, the fraction of the drug residing in the plasma is a determinant of drug elimination. When *V*_E_ is large, much of the drug is in extraplasmic space and is largely unavailable to feed any excretory organs. This is consistent with the herein observed increased *V*_E_ values associated with increased drug half-lives such that drug remained in the body for longer times.

For all four *V*_E_ values at *CL* of 100 and 40 ml/min of the families of curves shown, the time to achieve a high percentage of the terminal apparent volume of distribution and terminal half-life were longer for the GV models than for the E2 models (Table 1). In this study, the E2 models achieved their terminal apparent volumes of distribution as well as terminal half-lives of drug mass within 2 hours for *CL* of 100 ml/min and within 16 hours for *CL* of 40 ml/min. On the other hand, GV model had not been reached the terminal half-life of mass even within 24 hours following the intravenous bolus of drug for all *CL* and *V*_E_ values. The results in the Table 1 also show that GV model predicted longer terminal half-lives than the terminal half-lives from the E2 model. A sum of exponential terms model reduces to a monoexponential for late time, and monoexponentials inherit their rate constants from the local data region fit while GV model extrapolate a terminal function by inheriting only a shape from a local data region.

**Fig 4:**
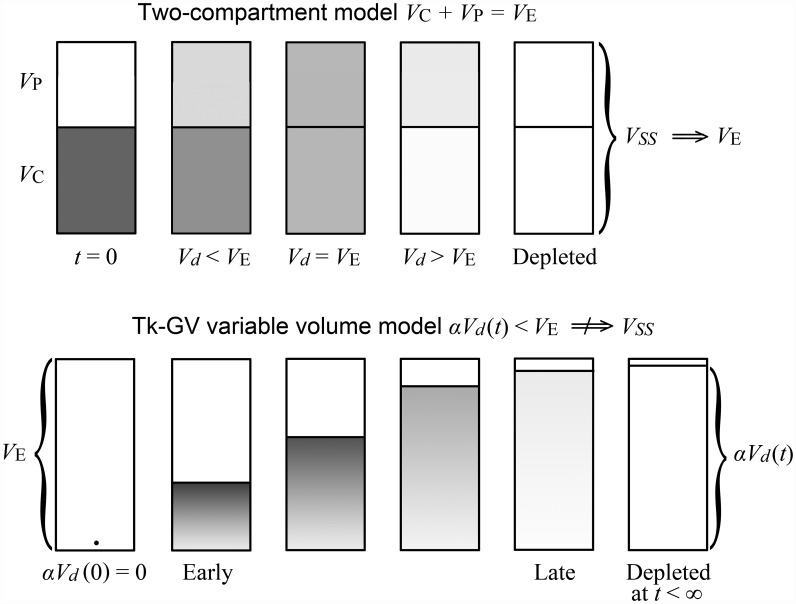
Schematic diagram showing E2 compartmental and GV variable volume models of drug distribution. The E2 model could also be drawn as a variable volume model in which case a scale factor *α*_exp_ = *V*_E_/*V*_d_(∞) < 1 would define the physical volume at time *t* to be *α*_exp_*V*_d_(*t*). Similarly, for the variable volume adaptively obtained GV model, one can define *α* = *V*_E_/*V*_d_(∞) < 1, and an expanding physical volume *αV*_*d*_(*t*). Note, both *α*_exp_ and *α* are constants at all times for their respective models. The term *V*_*SS*_ can be confusing because 1) *V*_*SS*_ implies that *V*_E_ is always a steady state volume, which is not the case as the GV model *αV*_*d*_(*t*) *< V*_E_ is concentration depleted at late time, see Eq (30). 2) *V*_*SS*_ implies that *V*_E_ only exists at *t* = ∞, whereas *V*_E_ is defined all of the time, i.e., on *t* = [0,∞) by Eqs (8 & 7). Finally, 3) *V*_*SS*_ implies an expected physical volume of distribution for sums of exponential term bolus models, and the apparent volume of distribution for a constant infusion experiment, whereas *V*_E_ applies to more models as the expected volume of physical distribution of a drug for both the bolus and constant infusion experiments.

Time samples of unmixed blood before arrival of a bolus in the sampling site are not used for washout modelling. Rather, the concentration fit function’s area under the curve of a washout model is obtained by back-extrapolation from peripheral vein samples obtained when vascular mixing is already advanced. The variable volume approach used here allows any number of summed exponential terms to be directly substituted into the single variable volume model without invoking compartments, and, the compartmental approach is merely another explanation for the same SET fit equations. The physical ambiguity for SET models is nothing new, for example, Niazi admixed mammalian compartmental and variable volume models without distinguishing between them and Zierler identified 10 different physical configurations for any E2 model [4,19]. Using a variable volume model, an apparent volume of uniform drug concentration that increases in time becomes possible. That is, the variable drug volume represents the apparent volume, containing drug at any particular time, which contrasts starkly with the corresponding compartmental concept of a constant *V*_area_, that only corresponds to the apparent volume of distribution at infinite time in a variable apparent volume of distribution model. In washout modelling, E2 model usage implies instant mixing of drug in the central compartment, generally composed of plasma and additional spaces into which the drug distributes extremely rapidly and therefore, E2 models predict a finite apparent volume of distribution at time zero. Subsequently, the drug distributes from the central compartment to the other body spaces, e.g., into a peripheral compartment, and drug elimination commences simultaneously with the drug administration. In E2 models, the distribution process completes after a few hours [20]. For a two-compartment model, there is always a time at which *V*_*d*_ transiently coincides with the value *Vss* and the further decrease of the plasma concentration of the drug is usually assumed to be only from drug elimination. The top of Fig 4 shows a schematic diagram of E2 model of drug distribution following bolus injection of a drug.

For GV models, the initial volumes of distribution are zero from Eq (26). The assumption of vanishingly small actual volume for the drug at the time of drug administration is realistic, *V*_*d*_ is approximately zero at time zero, after which the drug distributes throughout the body. Other evidence to this effect is that back-extrapolated early concentrations of ^51^Cr-EDTA, a GFR marker, have been observed to be more frequently of a linear logarithm of time shape, which functions set *C*(0) = *∞*, than of monoexponential shape, which do not [21]. Bottom Fig 4 shows a schematic diagram of GV model of drug distribution after bolus injection of a drug.

In prior work, in a 412 case series with both children and adults, some of us showed more precise and accurate *CL*-values for Tk-GV versus the less accurate E2 model compared to 8 h numerical integration controls for the slightly different *GFR* marker ^99m^Tc-DTPA [8]. In particular, the 4 h four sample E2 *CL*-values were significantly inflated by 4.9% compared to controls, and 4 h four sample Tk-GV *CL*-values were 0.5% (insignificantly) greater than eight sample, 8 h controls, for a net 4.2% *CL* difference between the 4 h E2 and Tk-GV experiments. In the current series, the 4 h, eight sample E2 *CL*-values were (significantly) 3.8% greater than the 4- h eight sample Tk-GV *CL*-values.

In this series, the expected volumes of distribution results were significantly less variable—see Tables 2 and 3—for Tk-GV than for E2. Indeed, for all the tested quantities, there were no result groupings for the Tk-GV parameters that were more variable than those for the E2 models, although there are many possible comparisons and admittedly not all possible parameter comparisons were calculated, and some tested differences were not highly significantly different. Thus, it would appear that in so far as we were able to test that assertion, the Tk-GV model PK parameter results were more precise than the E2 model. Given this finding and prior better accuracy results [8], the 5 to 9 times fold longer 95% of *V*_area_ times seen for the Tk-GV models are plausible. This may have important implications for future pharmacokinetic work.

## Conclusion

We have presented initial examples of the power of the general approach to variable volume modelling using a general time dependent volume of distribution model based on mass conservation that sets time varying drug body mass as proportional to volume of distribution. Terminal half-life depends on both expected drug distribution volume and the clearance. Furthermore, two commonly used model functions were used to illustrate behaviour of the general model. Exploration of other yet-undescribed fit function models and the modelling of drugs having more complicated pharmacokinetics is left for future work.
